# Detecting Rewiring Events in Protein-Protein Interaction Networks Based on Transcriptomic Data

**DOI:** 10.3389/fbinf.2021.724297

**Published:** 2021-09-08

**Authors:** Markus Hollander, Trang Do, Thorsten Will, Volkhard Helms

**Affiliations:** Center for Bioinformatics, Saarland Informatics Campus, Saarland University, Saarbrücken, Germany

**Keywords:** transcriptomics, domain-domain interaction (DDI), protein-protein interaction (PPI), isoform, alternative splicing (AS) events

## Abstract

Proteins rarely carry out their cellular functions in isolation. Instead, eukaryotic proteins engage in about six interactions with other proteins on average. The aggregated protein interactome of an organism forms a “hairy ball”-type protein-protein interaction (PPI) network. Yet, in a typical human cell, only about half of all proteins are expressed at a particular time. Hence, it has become common practice to prune the full PPI network to the subset of expressed proteins. If RNAseq data is available, one can further resolve the specific protein isoforms present in a cell or tissue. Here, we review various approaches, software tools and webservices that enable users to construct context-specific or tissue-specific PPI networks and how these are rewired between two cellular conditions. We illustrate their different functionalities on the example of the interactions involving the human TNR6 protein. In an outlook, we describe how PPI networks may be integrated with epigenetic data or with data on the activity of splicing factors.

## Introduction

Protein-protein interaction (PPI) networks are a popular cornerstone of integrative or computational cell biology and are frequently used to interpret the findings from high-throughput studies (Koh et al., 2012; Sevimoglu and Arga, 2014; Szklarczyk et al., 2019). Typically, PPI networks provide a genome-scale picture of all physical interactions detected between pairs of proteins. In the past, such networks have been compiled by integrating the results from many small-scale experiments and from several high-throughput experimental methods such as Yeast Two-Hybrid or Tandem Affinity Purification coupled to mass spectrometry (TAP-MS) (Bajpai et al., 2020). Full PPI networks provide a comprehensive picture of the interactome of the full proteome of an organism. However, in each cell at a particular moment in time, any physical protein-protein-contact can only be realized if both proteins are expressed at the same time. To address this, it has become common practice to trim general PPI networks to the set of proteins encoded by the genes that are expressed in the same condition. In this manner, researchers have compared the protein interaction landscape across tissues (Bossi and Lehner, 2009; Lopes et al., 2011) as well as the origin of tissue-specific diseases (Barshir et al., 2014).

PPI networks have an interesting scale-free topology, whereby highly connected “hub” proteins occur at a higher frequency than expected in, for example, a random graph. Furthermore, there exist densely connected communities (Girvan and Newman, 2002) of proteins participating in particular cellular functions or certain biological pathways. This ordering according to cellular function gives rise to a modular architecture of PPI networks. On a smaller scale, densely connected clusters are candidates for protein complexes and several algorithms exist to identify such complexes in PPI networks (Bader and Hogue, 2003; Nepusz et al., 2012; Will and Helms, 2014). Interacting partners and members of the same protein complex tend to be co-expressed (Jansen et al., 2002). The stable association of two proteins often involves one or more distinct structural contacts between specific domains of the proteins (Aloy and Russell, 2006). Knowledge about protein domain annotations and domain-domain interactions (DDIs) thus provides a good basis to describe protein associations (Aloy and Russell, 2006). DDIs were used, for example, to predict protein complexes and to analyze protein-protein interaction networks (Ozawa et al., 2010; Ma et al., 2012; Will and Helms, 2014).

About 95% of all human multi-exon genes are subject to alternative splicing (AS) (Pan et al., 2008) which clearly affects the ability of the encoded isoforms of the proteins to interact with other proteins (Buljan et al., 2012; Ellis et al., 2012). Hence, it appears worthwhile to exploit the base-resolution of modern RNA sequencing technology to resolve context-specific PPI networks at isoform-resolution. In 2015, the Vidal group published the first large-scale experimental study on isoform-specific protein interactions (Yang et al., 2016). They profiled the interactomes of 366 protein isoforms encoded by 161 genes and assayed them against a library of 13,000 genes. They found that accounting for isoforms gave a remarkable 3.2-fold increase of the number of PPIs. Strikingly, different isoforms of the same protein can interact with completely different proteins.

In the next section, we first give an overview of the numerous protein-level PPI databases that underpin the research effort in this field. These databases were recently reviewed in a comprehensive manner (Bajpai et al., 2020) and we will thus focus on a few popular meta webservices that offer integrated analyses and the ability to tailor full PPI networks to a particular cellular context. Afterwards, we present those tools and webservices in detail that support isoform-level analysis of protein interactions. Jalili and co-workers previously reviewed studies that integrated gene expression data with protein interaction networks (Jalili et al., 2018). Yet, their review focused on discovery of biomarkers and did not discuss the existing software tools, nor underlying domain models or protein isoform effects. Next, we review webservices and software tools that conduct differential comparisons of interactions between cellular contexts and thus facilitate the detection and study of PPI rewiring events. Finally, we will illustrate the usage and capabilities of some of them on the example of the interactions formed by the human TNR6 protein encoded by the FAS gene.

## Webservices Providing Protein-Level Data on Protein-Protein Interaction Networks

Manual and automated analyses of PPI networks require reliable and preferably large collections of PPIs. Consequently, many databases have been established over the years that collect, curate, and annotate protein interactions and make them available to the research community. These resources differ in their sources, curation and annotation policies, as well as their focus on, for example, particular species or interaction types, and the features of their interfaces.

Many of the available resources represent PPI networks at the level of integral genes, so that alternative splicing and protein isoforms are not considered. Already, the protein level enables powerful analyses of PPI rewiring between different conditions. Specifically, the human genome contains around 20,000 protein-coding genes (Pertea et al., 2018; Piovesan et al., 2019), while the human body consists of more than 200 different cell types (Bianconi et al., 2013). Each one of them will only express a cell-type specific subset of the full proteome, e.g. about 8,000–12,000 proteins (Dyring-Andersen et al., 2020). Hence, Bossi and Lehner argued that if two genes are co-expressed in a cell in a particular condition, their products may physically interact in that cell (Bossi and Lehner, 2009). However, if the two proteins are not simultaneously expressed in a tissue, then the interaction obviously cannot occur in that tissue. An examination of the relationship between tissue-specificity and connectivity found that proteins with more pronounced tissue-specificity are involved in fewer protein interactions than more universally expressed proteins (Bossi and Lehner, 2009). Furthermore, tissue-specific proteins are more likely to be recent evolutionary innovations than universally expressed proteins (Lehner and Fraser, 2004). Turned around, the more conserved a protein is, and the larger the number of tissues where it is expressed, the more protein interactions it is likely to have (Yeger-Lotem and Sharan, 2015). Filtering global PPI networks to the subset of expressed genes or proteins thus became the workhorse for generating tissue- and other context-specific PPI networks.

Table 1 presents an overview of major primary and meta PPI databases and their features. Most of the resources discussed here provide a web-interface that enables users to query and download the underlying interaction data. In many cases this includes integrated visualization of the queried interactions. The subsequent sections discuss a few meta databases in more detail whose webservices additionally offer PPI (sub-)network analyses or processing of user provided data in a context-specific manner.

**TABLE 1:** Overview of PPI databases and PPI network (PPIN) features of their webservice.

	Data collection	Source type	Species	Webservice	Context filter	Visual-ization	PPIN analyses
APID	Meta	Evidence	Multiple	apid.dep.usal.es	No	Yes	No
BIND	Primary	Evidence	Multiple	—	—	—	—
BioGRID	Primary	Evidence	Multiple	thebiogrid.org	No	Yes	No
DIP	Primary	Evidence	Multiple	dip.doe-mbi.ucla.edu	No	Yes	No
HIPPIE	Meta	Evidence	Human	cbdm.uni-mainz.de/hippie	Tissues, diseases, functional	Yes	Enrichment
HPIDB	Both	Evidence, predicted	Multiple	hpidb.igbb.msstate.edu	No	Yes	No
HPRD	Primary	Evidence	Human	hprd.org	No	No	No
HuRI	Primary	Evidence	Human	interactome-atlas.org	Tissued	Yes	No
I2D	Meta	Evidence, predicted	Multiple	ophid.utoronto.ca	No	No	No
IID	Meta	Evidence, predicted	Multiple	iid.ophid.utoronto.ca	Tissues, druggability, localization, diseases	Yes	Enrichment, topology
InnateDB	Both	Evidence, predicted	Multiple	innatedb.com	Tissues, cell types, diseases	Yes	Topology
IntAct	Both	Evidence	Multiple	ebi.ac.uk/intact	No	Yes	No
iRefWeb	Meta	Evidence, predicted	Multiple	wodaklab.org/iRefWeb	No	No	No
MatrixDB	Both	Evidence, predicted	Multiple	matrixdb.univ-lyon1.fr	Tissues	Yes	No
mentha	Meta	Evidence	Multiple	mentha.uniroma2.it	No	No	Paths
MINT	Primary	Evidence	Multiple	mint.bio.uniroma2.it	No	Yes	No
MPact	Primary	Evidence	Yeast	—	—	—	—
MPIDB	Both	Evidence	Microbes	—	—	—	—
MPPI	Primary	Evidence	Mammals	mips.gsf.de/proj/ppi	No	No	No
MyProteinNet	Meta	Evidence	Multiple	netbio.bgu.ac.il/myproteinnet2	Tissue, expression, single cell	Yes	No
PIP	Meta	Evidence, predicted	Human	compbio.dundee.ac.uk/www-pips	No	No	No
PrePPI	Meta	Evidence, predicted	—	bhapp.c2b2.columbia.edu/PrePPI	No	Yes	No
SPECTRA	Meta	Evidence	Human	alpha.dmi.unict.it/spectra	Tissue, tumors, expression	Yes	Network alignments
STRING	Meta	Evidence, predicted	Multiple	string-db.org	No	Yes	Enrichment, clustering
TissueNet	Meta	Evidence	Human	netbio.bgu.ac.il/tissuenet	Tissue, expression	Yes	No

### Protein-Protein Interaction Databases

Interaction resources can be broadly categorized as primary databases, which collect and curate data independently, or meta databases, which compile their data from primary databases. All resources discussed here focus on experimentally verified interactions, typically gathered from publications and submissions, though a few also incorporate predicted interactions.

Some of the earliest established primary PPI resources that cover a wide range of species are the Biomolecular Interaction Network Database (BIND) (Bader et al., 2003), the Database of Interacting Proteins (DIP) (Salwinski et al., 2004), the Molecular Interaction Database (MINT) (Licata et al., 2012), and IntAct (Orchard et al., 2014). Other primary databases concentrate their curation efforts on a particular species or a subset of species. For instance, the MIPS Mammalian Protein-Protein Interaction Database (MPPI) (Pagel et al., 2005) collects mammalian PPIs, while MPact (Güldener et al., 2006) focuses on yeast and the Microbial Protein Interaction Database (MPIDB) (Goll et al., 2008) on microbial PPIs. The Human Protein Reference Database (HPRD) (Prasad et al., 2009) and the Human Reference Protein Interactome Mapping Project (HuRI) (Luck et al., 2020) are prominent databases centered around human interactions.

Furthermore, there are resources that specialize in types or subsets of interactions. The B Cell Interactome (BCI) (Lefebvre et al., 2007) concerns itself with physical molecular interactions in human B cells. Similarly, InnateDB (Breuer et al., 2013) compiles and predicts interactions involved in the innate immune response to microbial infections in humans, mice, and bovines. MatrixDB (Huang et al., 2018) concentrates on interactions of proteins, proteoglycans, and polysaccharides of the extracellular matrix. While most resources deal with intra-species interactions, the Host-Pathogen Interaction Database (HPIDB) (Ammari et al., 2016) gathers and predicts inter-species molecular interactions between pathogens and their hosts.

To establish common standards among the major interaction resources, the International Molecular Exchange (IMEx) Consortium was founded in 2005 (Orchard et al., 2012; Porras et al., 2020). Of the primary databases discussed here, current members include BIND, DIP, HPIDB, InnateDB, IntAct, MatrixDB, and MINT, whereas MPact, and MPIDB contributed in the past but are no longer active members. The IMEx Consortium developed a set of curation rules for physical molecular interactions extracted from publications and maintains a set of uniquely defined molecular identifiers for these interactions for consistency. The non-redundant interactions compiled by this joined curation effort are available through the IMEx Consortium and IntAct, and members can include all interactions or a suitable subset in their own databases.

A major primary interaction database that is not part of the IMEx Consortium is the Biological General Repository for Interaction Datasets (BioGRID) (Oughtred et al., 2021). BioGRID focuses on genetic, chemical and protein interactions as well as post translational modifications curated from individual studies and high-throughput datasets for many species. It has substantial overlap with the data from the IMEx Consortium (Porras et al., 2020) and high coverage of PPIs from primary sources in general (Bajpai et al., 2020).

In addition to primary databases, there exists an increasing number of meta resources that compile and provide access to PPI data from multiple primary sources. The Interologous Interaction Database (I2D) (Brown and Jurisica, 2007) conducts some manual curation of interactions for model organisms from the literature but mainly combines data from primary databases such as BCI, BIND, BioGRID, DIP, HPRD, InnateDB, IntAct, and MINT with predicted interactions. Similarly, the Protein-Protein Interaction Prediction (PIP) database (McDowall et al., 2009) supplements human PPI data from BIND, DIP, I2D, and HPRD with predicted PPIs, while the PrePPI database (Zhang et al., 2013) adds predicted interactions to experimental data from BioGRID, DIP, HPRD, IntAct, MINT, and MPPI. A meta database for evidence based interactions in model organisms is mentha (Calderone et al., 2013) that uses BioGRID, DIP, IntAct, MatrixDB, and MINT as sources. The Agile Protein Interactomes DataServer (Alonso-López et al., 2019) unifies interactions from BioGRID, DIP, HPRD, IntAct, and MIND, while iRefWeb (Turinsky et al., 2021) consolidates PPI data from BIND, BioGRID, DIP, HPRD, I2D, IntAct, MINT, MPact, and MPPI.

However, some primary databases may also incorporate data from one or more of the other primary databases in addition to their own collected data. For example, MPIDB includes interactions from BIND, DIP, IntAct, and MINT, while HPIDB integrates data from BIND, BioGRID, DIP, I2D, InnateDB, IntAct, MatrixDB, and MINT. Typically, this involves extracting a subset of interactions relevant to the more specialized focus from the other databases. Notably, IntAct provides the infrastructure for other curators and resources to enter and annotate data into their database, and hosts and provides access to the complete data curated by the IMEx Consortium (Orchard et al., 2014). A recent systematic review of PPI databases provides a visual overview of the intertwined dataflow between the various primary and meta databases (Bajpai et al., 2020).

### Search Tool for Retrieval of Interacting Genes/Proteins (STRING)

The Search Tool for Retrieval of Interacting Genes/Proteins (STRING) (Szklarczyk et al., 2019) is a widely used meta database and webtool for the retrieval and analysis of evidence based and predicted PPIs. STRING merges all curated interactions from the IMEx Consortium (Porras et al., 2020) and BioGRID (Oughtred et al., 2021), and combines them with manually curated interactions from pathway records collected by the KEGG (Kanehisa et al., 2021), Reactome (Jassal et al., 2020), BioCyc (Karp et al., 2019), and Gene Ontology (Carbon et al., 2021) databases. These PPIs are supplemented with text-mining and predictions based on genomic context, gene co-expression and protein co-expression. Interactions between two proteins are scored in the interval [0,1] according to the estimated confidence of the interaction. In total, STRING (v11.5) contains almost 300 million interactions of the highest confidence (STRING confidence score ≥0.9) for 67 million proteins of over 14,000 organisms, which makes it the most comprehensive tool in terms of coverage of proteins and organisms (Szklarczyk et al., 2019; Bajpai et al., 2020).

Users can get access to evidence-based and predicted PPIs through a user-friendly web-interface, where an expansive list of associated studies and detailed annotations from other databases is available, together with sub-network visualization, enrichment and clustering analyses that are ready for ad-hoc uses. STRING can be queried for single or multiple proteins, protein families, or organisms. Alongside the query proteins, users can optionally provide additional information such as abundance, fold change, or *p*-values to be considered by the functional enrichment analysis. However, thus far STRING does not provide an option to filter for different tissues or to a user-provided expression data set.

As part of the ELIXIR core data resources (Crosswell and Thornton, 2012), STRING provides known and predicted PPIs of high confidence for incorporation with other well-maintained biological databases that serve as a foundation for analysis services in the ELIXIR ecosystem. Moreover, STRING can be found in the underlying architecture of various databases and analysis methods, such as HAPPI (Chen et al., 2009), a compilation of human protein interactions, or RegNetwork (Liu et al., 2015), a database for transcriptional and post-transcriptional networks in human and mouse, or GSA-SNP2 (Yoon et al., 2018), a tool for pathway enrichment and network analysis, or in some PPI-analyzing tools like PPIXpress (Will and Helms, 2016). Furthermore, the use of STRING as a reference database for large-scale analysis and profiling of proteomes is extremely common as well. One example for such a use-case is the characterization of protein subnetworks of biomarkers specific for human diseases using STRING PPI scores for the accurate prediction of pathway-affected gene drivers (Liang et al., 2016).

### Human Integrated Protein-Protein Interaction Reference (HIPPIE)

The Human Integrated Protein-Protein Interaction Reference (HIPPIE) (Alanis-Lobato et al., 2017) is a meta database and webservice that facilitates access to and context-specific analysis of experimentally detected human PPIs. These interactions are consolidated from BIND (Bader et al., 2003), BioGRID (Oughtred et al., 2021), DIP (Salwinski et al., 2004), HPRD (Prasad et al., 2009), IntAct (Orchard et al., 2014), MINT (Licata et al., 2012), and MPPI (Pagel et al., 2005). HIPPIE assigns confidence scores to the PPIs based on the quality and reliability of the experiments supporting them. The tissue specificity of the PPIs is derived from tissue RNAseq data from the GTEx Consortium (Lonsdale et al., 2013), and they are further annotated with functional information from the Gene Ontology (Carbon et al., 2021).

**FIGURE 1:**
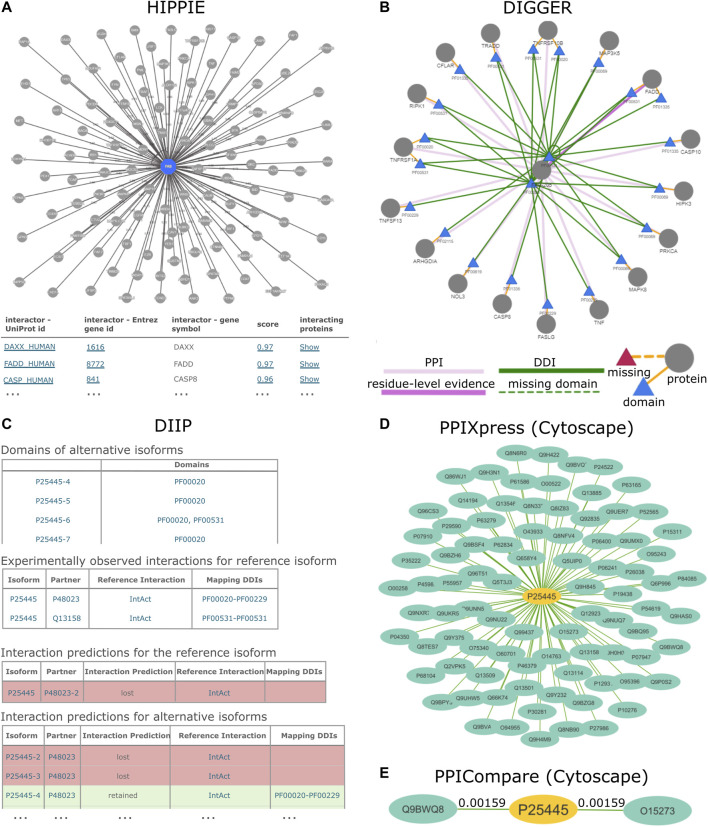
PPI results for the human TNR6 protein encoded by the FAS gene (UniProt accession: P25445) from HIPPIE, DIIP, DIGGER, PPIXpress and PPICompare. **(A)** HIPPIE presents general interacting partners as a PPI network view and a table with evidence scores. **(B)** DIGGER presents an interactive PPI-DDI view that combines different aspects of a PPI for a single isoform. The domain and exon architecture view and more detailed results for individual interactions, domains and isoforms are not shown here. **(C)** DIIP compiles tables with the domains and predicted interaction retention for alternative isoforms. **(D)** PPIXpress was applied to RPKM expression data of neuronal stem cells and H1 stem cells from the Roadmap Epigenomics project (Kundaje et al., 2015). Shown here are the interaction partners of TNR6 in the resulting neuronal stem cell specific PPI network. **(E)** PPICompare was subsequently applied to cell-specific PPIs generated by PPIXpress to produce the differential PPI network between the two cell types. Illustrated here are two interaction gains in the transition from H1 stem cells to neuronal stem cells with the respective adjusted *p*-value. The graphics for the subnetworks from PPIXpress and PPICompare were generated with Cytoscape.

The web-interface enables users to query the database with individual proteins or PPI networks in a tissue- and function-specific manner, with the option to define a custom context and to specify the desired interaction types and level of confidence. HIPPIE subsequently generates and visualizes a query-specific PPI network (Figure 1A) and can optionally perform edge direction inference, prediction of inhibitory or activating effects, and enrichment analysis of disease, process, function and cellular compartment annotations. In addition to the web-interface, HIPPIE offers an application programming interface (API) that facilitates automated queries and thus integration into analysis pipelines.

The integration of experimentally confirmed PPIs from expert-curated sources makes HIPPIE a reliable and resourceful reference database to be employed in various scenarios. Many studies availed the tool to collect high confidence PPIs to support their study hypothesis, verify experimental results or control the quality of analytic methods. Some of such use-cases include the work by Sundell et al., who assessed the performance of phosphomimetic proteomic peptide-phage display in detecting ligands of short linear motifs by comparing the identified ligands to those reported by HIPPIE (Sundell et al., 2018), and the work by Kruse et al., who screened for candidate protein constituents of N-cadherin complexes based on HIPPIE PPI confidence scores (Kruse et al., 2019). Furthermore, the tool is widely employed to analyze pathogenesis or developmental processes where tissue-specificity has a substantial weight on defining the PPI networks. This is well illustrated by a comparative study by Verma et al. where the subtle differences in LRRK2 interactomes across different brain subregions, kidney and lung could be spotted using HIPPIE’s tissue filter (Verma et al., 2021). On the other hand, this webservice is often used as a meta-database and integrated in other more context-specific analysis methods such as GSA-SNP2 (Yoon et al., 2018), PSSMsearch (Krystkowiak et al., 2018), or LncDEEP (Yang et al., 2018).

### Integrated Interactions Database (IID)

The Integrated Interactions Database (IID) from the Jurisica lab (Kotlyar et al., 2019) is a database and webtool that offers full PPI networks of several species, context-specific sub-networks, as well as topology and enrichment analyses. IID integrates experimentally detected protein interactions from the following 9 curated databases: BCI (Lefebvre et al., 2007), BIND (Bader et al., 2003), BioGRID (Oughtred et al., 2021), DIP (Salwinski et al., 2004), HPRD (Prasad et al., 2009), InnateDB (Breuer et al., 2013), IntAct (Orchard et al., 2014), I2D (Brown and Jurisica, 2007), and MINT (Licata et al., 2012). Additionally, it includes orthologous PPIs and computationally predicted interactions. Overall, it provides interactions for human (*H. sapiens*), the 5 model organisms fly (*D. melanogaster*), mouse (*M. musculus*), rat (*R. norvegicus*), worm (*C. elegans*), and yeast (*S. cerevisiae*), and for 12 domesticated species.

Given a set of query proteins, IID generates a sub-network of either all interactions involving the query proteins or only interactions between the query proteins. Users can specify the desired level and type of evidence supporting the PPIs. The resulting sub-network can be further pruned in a context-specific manner to selected tissues, sub-cellular localizations, diseases, druggability status, or any combination thereof. Moreover, IID can analyze the network topology in terms of node degree, clustering coefficient and betweenness, as well as the enrichment of diseases, tissues, sub-cellular localizations and druggability in the network PPIs. All results are displayed as tables and can be downloaded as such with customizable annotations. While the web-interface does not include network visualization, the result files are compatible with Cytoscape (Shannon et al., 2003).

As a consequence of the ease of use combined with the number of context-specificity options, IID is a flexible resource that can be of use in a wide range of applications. For example, in a recent study, protein interactions from IID combined with a transcription regulatory network suggested the Hippo-signaling pathway mediator TAZ as a regulator of numerous metabolic genes and thus as a link between tension sensing and dendritic metabolic programming, which could then be corroborated by TAZ knock-down in mice (Chakraborty et al., 2021). Investigating the effect of mutations in key autophagy proteins in cancer, IID was employed to construct the interactomes of the autophagy kinase ULK1 (Kumar and Papaleo, 2020) and the autophagosome protein LC3B (Fas et al., 2020). Furthermore, PPI data provided by IID is used by resources such as pathDIP (Rahmati et al., 2020), which predicts physical pathway associations for proteins based on physical species-specific protein interactions, or the interactive online platform CoVex (Sadegh et al., 2020), which facilitates exploration of the SARS-CoV-2 host interactome.

### MyProteinNet and TissueNet

MyProteinNet (Basha et al., 2015) and TissueNet (Basha et al., 2017) are two webservices for context-specific PPI networks developed by the Yeger-Lotem lab. With TissueNet, users can retrieve tissue-specific PPIs for a single human query protein or protein interaction. The underlying human PPI data is compiled from experimentally validated physical protein interactions retrieved from BioGRID (Oughtred et al., 2021), DIP (Salwinski et al., 2004), IntAct (Orchard et al., 2014), and MINT (Licata et al., 2012). The tissue-specificity is computed based on tissue expression profiles obtained from GTEx (Lonsdale et al., 2013) and the Human Protein Atlas (Uhlén et al., 2015). TissueNet displays the expression of the query protein and its interactors for all available tissues as well as associated Gene Ontology annotations, and the integrated network viewer allows users to switch between tissues on the fly. The underlying tissue-specific PPI networks can be downloaded and have proven useful in the study of, for example, schizophrenia risk genes (He et al., 2021), the neurological effects of COVID-19 (Prasad et al., 2021), and susceptibility pathways among various cancers (Qian et al., 2015).

In contrast, MyProteinNet focuses on building customizable interaction networks for humans and other model species. Based on a user-selected organism and combination of PPI databases to be used, MyProteinNet assembles a general network of experimentally validated physical protein interactions. In addition to BioGRID, DIP, IntAct and MINT, users can choose to include PPIs from InnateDB (Breuer et al., 2013), MatrixDB (Huang et al., 2018), and STRING (Szklarczyk et al., 2019), among others. This step offers users the option to add their own interaction data, and even to only use user-provided interactions for network construction. Furthermore, MyProteinNet accepts single-cell, tissue, and other expression data to prune the general PPI network in a context-specific manner. For humans, the tissue-specific expression data used by TissueNet can be applied for filtering without requiring additional uploads by the user. The results consist of a file with the global, unfiltered interactome, a file with the context-specific interactome, and a file with the PPIs removed during the pruning step. These network files can be visualized with Cytoscape (Shannon et al., 2003).

The customizable nature of the context-specific PPI networks generated by MyProteinNet makes it flexible in its application. For instance, it has been used by the Yeger-Lotem lab to analyze and highlight human tissue-selective processes and genetic disorder genes (Basha et al., 2020). In other studies, it served, for example, as the basis for identifying tissue-specific driver proteins in human interactomes (Zhang et al., 2016), or for examining the functional centrality of cancer genes in comparison with genes involved in other diseases (Ostrow and Hershberg, 2016).

## Resources and Software Tools on Domain-Level and Isoform-Level Protein-Protein Interaction Data

A few approaches that model context-specific PPI networks utilize an underlying domain model in which each protein is represented by one or more structural domains. Using such a domain model incorporates elements of three-dimensional protein structures. As domain-domain interactions (DDIs) tend to be evolutionary conserved (Itzhaki et al., 2006), experimental evidence on domain interactions can be transferred between related organisms. Isoform-specific expression data enables detecting effects of alternative splicing at the level of full protein domains. Hence, DDI-based tools allow predicting the interactomes of specific protein isoforms in a particular condition or tissue. Table 2 presents a feature overview of such webservices and software tools.

**TABLE 2:** Features of software tools and webservices enabling the generation and analysis of domain- or isoform-level protein-protein interaction data (DIIP, DIGGER, PPIXpress), as well as the comparison of interaction rewiring (SPECTRA, DifferentialNet, PPICompare).

	DIIP	DIGGER	PPIXpress	SPECTRA	DifferentialNet	PPICompare
**Type**	Webservice	Webservice	Stand-alone tool	Webservice	Webservice	Stand-alone tool
**Species**	Human	Human	Multiple	Human	Human	Multiple
**Resources**						
PPI	HI-II-14, IntAct	BioGRID	IntAct, mentha	BioGRID, HPRD, MIPS, IntAct	BioGRID, DIP, IntAct, MINT	PPIXpress
DDI	3did, DOMINE	3did, DOMINE	DOMINE, IDDI, 3did, iPfam	Not available	Not available	PPIXpress
Other	Pfam (domain annotations)	PDB (exon-specific residues)	UniProt + Ensembl (protein details), Pfam-A	Protein Atlas, ArrayExpress, GEO, TCGA (tissues, tumors)	GTEx, Human Protein Atlas (tissues)	Ensembl (protein details)
**Query type**						
Protein query	Single gene or protein (UniProt, HGNC)	Single gene, transcript, protein (Ensembl, HGNC)	Not available	All genes in SPECTRA	Up to 5 genes or proteins (Entrez, Ensembl)	Not available
		Single exon (Ensembl, HGNC, coordinates)		List of genes (Entrez, Ensembl, UniProt)		
				Gene expression		
Network query	Single protein and list of interacting partners	List of isoforms, transcripts, genes	Gene or transcript expression data (UniProt, HGNC, Ensembl IDs)	SPECTRA	Not available	Two sets of condition-specific PPI and DDI networks (PPIXpress)
		Transcript expression counts	Reference PPI network (optional)	PPI network(s) for comparison		
**Integrating output from other tools or databases**	Not available	Expression data: Cufflinks, Kallisto, count matrix	PPI networks: mentha, IntAct, BioGRID, HPRD, custom PPI list	Expression matrix	Not available	PPI and DDI networks: PPIXpress
			Expression data: Cufflinks, Kallisto, (TCGA) RSEM, GENCODE GTF, count matrix			
**Output**	Isoform domains	Isoform and exon domains	Context-specific PPI and DDI network(s)	Context-specific PPI network	Tissue-specific differential PPIs	Context-specific differential PPIN network
	PPI prediction for isoforms	PPIs and DDIs for isoforms and exons		Differential PPI subnetworks		Minimal set of rewiring causes
**Features**						
Visualization	Not available	Integrated (protein, domain, and interaction view)	Network output available for Cytoscape	Integrated	Integrated	Network output available for Cytoscape
Context-specific	Not available	User-defined	User-defined	Tissues, tumors, user-defined	Tissues	User-defined
Interaction scoring	DDI-based loss/retention prediction for isoforms	DDI-based missing interactions for isoforms	DDI-based presence for main isoform	PPIs: coverage, average weight	Difference between tissue-specific and median expression score	Significance of PPI rewiring event
				Differential subnetworks: expression log fold change		
Batch analysis	Not available	Not available	Yes	Not available	Not available	Yes

### Domain-Domain Interaction Databases

Domain- and isoform-level methods tend to use Pfam domains (Mistry et al., 2021), which are typically identified in experimentally determined three-dimensional structures of protein complexes by assessing whether neighboring domains are in close contact. This information is compiled by web services such as iPfam (Finn et al., 2014) and 3did (Mosca et al., 2014). The database 3did is regularly updated and currently contains 14,972 DDIs for 9,580 domain families in its current release (Pfam version 32.0, PDB version 2020_05). Furthermore, the structurally derived domain-level interactome can be enriched by computational predictions of DDIs between domain families (Riley et al., 2005; Guimarães et al., 2006). The meta database DOMINE (Yellaboina et al., 2011) integrated two databases of PDB-derived DDIs and 7 predicted data sources. Moreover, IDDI (Kim et al., 2012) combined data from three structure-based DDI sources and 20 computational datasets.

### DomainGraph

A tool enabling users to inspect the effect of AS on interaction networks is the Cytoscape 2.x (Shannon et al., 2003) plugin DomainGraph (Emig et al., 2010). When combined with the AS analysis tool AltAnalyze (Emig et al., 2010), DomainGraph visualizes those protein domains in DDI networks that are affected by differential exon usage. Alternatively, users can submit a gene or PPI network to DomainGraph to visually inspect the interactions between the genes, their associated protein isoforms and underlying DDIs, with the option to highlight putatively AS-affected network components if exon expression data is available. This tool is made for interactive use whereby a user can manually investigate the implications of associated changes in the PPI and DDI networks. However, the tool is not able to automatically process proteome-scale PPI networks.

Thus far, DomainGraph has been used primarily to visualize differential components identified by AltAnalyze, such as alternative exons between normal and cancerous colorectal tissues (Aziz et al., 2014), alternatively spliced transcripts between normal and degenerated macula (Whitmore et al., 2013), or alternative protein domains and miRNA binding sites between herpes-infected and noninfected lymphatic epithelial cells (Chang et al., 2011).

### Domain-Based Isoform Interactome Prediction (DIIP)

Domain-based Isoform Interactome Prediction (DIIP) (Ghadie et al., 2017) is a method that uses reference PPIs and DDIs to predict isoform interactions, which can be queried with the accompanying webservice (https://predict-isoform-interactome.herokuapp.com). Given a human query protein, the DIIP webservice lists the domains in alternative isoforms, experimentally observed PPIs involving the reference isoform, as well as interaction predictions for alternative isoforms (Figure 1B).

To construct the underlying human isoform interactome, DIIP first builds a PPI network with experimentally determined PPIs from the HI-II-14 dataset (Rolland et al., 2014) and the IntAct database (Orchard et al., 2014). The network proteins are annotated with Pfam domains (Mistry et al., 2021), which then guide the mapping of DDIs from 3did (Mosca et al., 2014) and DOMINE (Yellaboina et al., 2011) onto the PPIs. Finally, DIIP predicts isoform interactions based on the presence of interacting domains in alternative isoforms retrieved from UniProt (Bateman et al., 2021). A PPI between two isoforms is considered lost if none of the supporting DDIs can be realized due to the required domains missing from the isoforms. Otherwise, the PPI is predicted to be retained.

In the accompanying study, the authors showed that alternative splicing is responsible for extensive network remodeling of protein interactions (Ghadie et al., 2017). For about 22% of the genes with two or more isoforms in the predicted isoform interactome, at least one isoform lost an interaction. Furthermore, different interaction profiles were found for roughly 18% of the isoform pairs encoded by the same gene in the isoform interactome.

### PPIXpress

**FIGURE 2:**
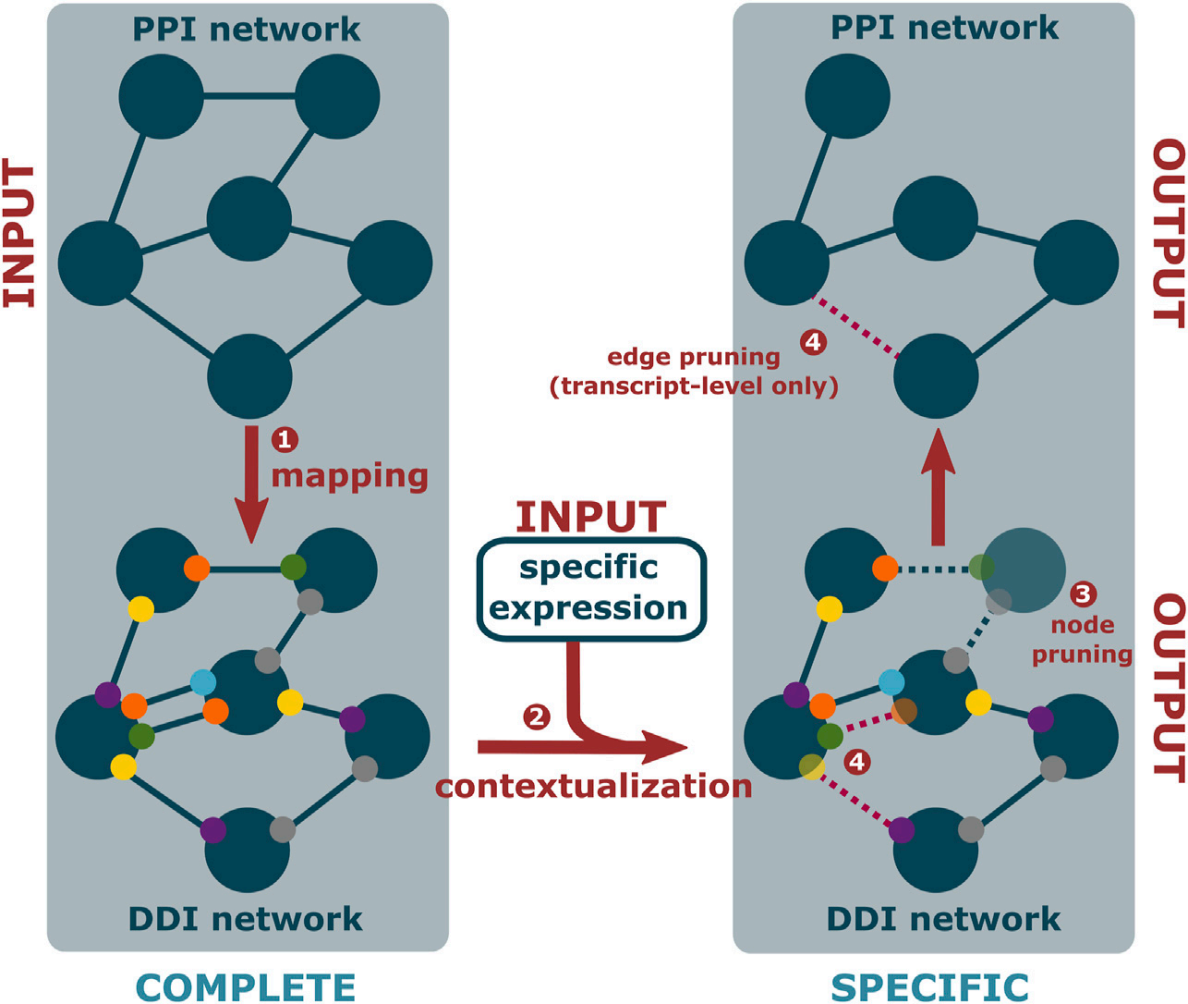
PPIXpress first takes a complete PPI network (dark blue nodes) and associates each interaction (solid edges) with known interactions between protein domains (smaller, colorful nodes) or, if unknown, between artificial domains (grey nodes) (1). Then, based on provided expression data (2), proteins that are not expressed and domains that do not occur in the most abundant transcript are removed (translucent) (3), as are the no longer supported edges (dotted lines) if transcript-level data is available (4), resulting in a condition-specific DDI and corresponding PPI network.

PPIXpress (Will and Helms, 2016) is a stand-alone tool developed in the Helms group that constructs condition- or sample-specific protein-protein interaction networks from transcriptomic data. It considers underlying domain-domain interactions and can be applied on the gene-level as well as the transcript-level, thereby capturing the effects of alternative splicing in addition to those of differential expression. As outlined in Figure 2, the approach consists of a mapping step that relates protein-protein interactions to domain-domain interactions and a contextualization step that removes domain-domain interactions not supported by the given expression data, yielding a condition-specific PPI network (Figure 1D).

#### Condition-Specific Domain-Domain Interaction and Protein-Protein Interaction Network Construction

Besides one or more gene- or transcript-level expression data samples provided by the user, PPIXpress requires a reference PPI network with condition-unspecific interactions from the corresponding species. This reference network can be a custom one supplied by the user or can be automatically retrieved from the current versions of the databases mentha (Calderone et al., 2013) or IntAct (Orchard et al., 2014). The STRING database (Szklarczyk et al., 2019) can be queried to add functional association scores. The most recent Ensembl (Howe et al., 2021) and UniProt (Bateman et al., 2021) databases are queried for gene and transcript annotations, which are subsequently associated with Pfam-A domains (Mistry et al., 2021) by InterProScan (Jones et al., 2014). For physical domain-domain interaction data, a pre-compiled database of high-confidence data from DOMINE (Yellaboina et al., 2011) and IDDI (Kim et al., 2012) is used, which is supplemented by automatically retrieved data from the iPfam (Finn et al., 2014) and 3did (Mosca et al., 2014) databases.

The initial mapping stage (Figure 2, step 1) starts from the complete, condition-unspecific PPI network. Each interaction therein is then annotated with known interactions between domains in the longest isoform of the participating proteins. To ensure complete correspondence of the resulting domain-domain interaction network and the PPI network, artificial domains are added to interacting proteins if their interaction cannot be assigned to at least one domain-interaction. These artificial domains counteract the sparsity of domain-level data, where protein-protein interaction may not be accounted for by a known domain-domain interaction. In a similar previous approach, this improved the performance of protein complex prediction without negatively affecting precision (Ma et al., 2012).

In the subsequent contextualization stage (Figure 2, step 2), node pruning (Figure 2, step 3) is conducted by removing proteins from the network that are not supported by sufficient gene or transcript expression in the sampled condition, similar to established gene-based approaches. When PPIXpress is used on the gene-level, each protein is represented by its longest isoform just like in the initial mapping. Due to the direct correspondence between the two layers, pruning then stops here. When applied on the transcript-level, however, there is an additional edge pruning step (Figure 2, step 4), in which the protein-protein interactions that are not backed by at least one domain-domain interaction in the most abundant transcript are trimmed as well. In both cases, the result is a pruned, condition-specific PPI network annotated with the participating domain-domain interactions.

#### Applications: Protein-Protein Interaction Rewiring in Cancer

The accompanying case study (Will and Helms, 2016) applied PPIXpress to 112 matched breast cancer and healthy tissue samples obtained from The Cancer Genome Atlas (Weinstein et al., 2013) and constructed a single differential PPI network of significantly rewired interactions between the two conditions from the combined individual condition-specific networks. The rewired interactions were associated with hallmarks of cancer if at least one of the proteins was annotated with a corresponding Gene Ontology (GO) (Carbon et al., 2021) term or KEGG (Kanehisa et al., 2021) pathway, and in addition GO term enrichment analysis was performed. To compare the performance, this approach was conducted with PPIXpress set to the gene-level and then to transcript-level filtering.

Although the gene-based approach generated larger networks, it detected fewer significant changes in interactions between the tumor and healthy samples. In contrast, the transcript-based network construction found significantly more rewiring events associated with the hallmarks of cancer in the differential network. Additionally, enriched KEGG and GO terms were related to carcinogenic processes, and the transcript-level differential network contained more enriched KEGG pathways and GO biological processes. Overall, the inclusion of domain-level and transcript-level information improved the performance and statistical significance of the results.

Frishman and colleagues used PPIXpress to generate 642 patient-specific pairs of interactomes corresponding to both the tumor and healthy tissues across 13 cancer types based on RNA-Seq datasets from The Cancer Genome Atlas (Kataka et al., 2020). The underlying hypothesis for this study was that isoform switching has been noted as a hallmark of cancer. Isoform switching often results in the loss or gain of domains mediating protein interactions and thus, the re-wiring of the interactome. Comparison of these interactomes between tumor and normal samples gave a list of patient-specific “edgetic” perturbations of the interactomes associated with the cancerous state. Interestingly, the majority of the rewiring events did not directly affect significantly mutated genes but were nonetheless strongly correlated with patient survival. The findings of this study are made available as EdgeExplorer: http://webclu.bio.wzw.tum.de/EdgeExplorer. The involved proteins are suggested both as a new source of potential biomarkers for classifying cancer types and as putative anti-cancer therapy targets.

### Domain Interaction Graph Guided Explorer (DIGGER)

The Domain Interaction Graph Guided Explorer (DIGGER) (Louadi et al., 2021) is a webservice (https://exbio.wzw.tum.de/digger) that leverages domain- and residue-level information to expedite studying the mechanistic effects of alternative splicing in humans. For domain-level analysis, DIGGER constructs a joint interaction network of human PPIs retrieved from BioGRID (Oughtred et al., 2021) and accompanying DDIs selected from 3did (Mosca et al., 2014) and DOMINE (Yellaboina et al., 2011). With this data structure, the mapping of PPIs and DDIs to exon- and transcript-defined regions is facilitated for higher-leveled analysis of the interactomes. First, residues from experimentally resolved protein structures from the Protein Data Bank (PDB) (Burley et al., 2021) are aligned with the amino acid residues from a protein in the joint network. Then, the interactions between the mapped resolved residues from different isoforms are used as evidence for the interactions between the domains in the isoforms. This allows DIGGER to locate the exons or transcripts associated with these domains using the mapping positions. Since a single amino acid residue or domain might be involved in the interaction between a protein and multiple partners, the authors defined an interaction scoring scheme based on the fraction of annotated DDIs present between two proteins. Finally, based on the score gradient one can assess to which degree a PPI is affected and draw inference on underlying mechanisms that impact those interactions, such as exon skipping. Notably, DIGGER combines all structural information from different isoforms of the same gene, whereas other tools such as PPIXpress (Will and Helms, 2016) consider only a particular transcript, typically the most strongly expressed one.

The webservice offers three different use modes that can be used interchangeably. In the isoform-level analysis, the user can comprehensively visualize the interacting domains of proteins and compare the interactions of different isoforms (Figure 1C). In addition to displaying the associated PPIs, this mode visualizes the interactions annotated with a particular domain using the underlying protein and domain interaction data. Furthermore, each domain in the isoform can be selected to show a domain-centered interaction view. Lastly, this mode offers an overview of the domain and exon architecture of the selected isoform. The exon-level mode is similar and focuses on a particular exon. In the network-level analysis, the user can explore interactions between multiple isoforms and generate a specific subnetwork from a list of protein variants or transcripts. Here, the user can optionally upload a particular expression data set with transcript counts, thus accounting for user-defined contexts.

In their paper, Louadi et al. demonstrated how DIGGER can be used to confirm the effects of alternative splicing on PPIs and DDIs (Louadi et al., 2021). A case study where the PPI networks for two ALK transcripts were visualized using DIGGER revealed that 97% of the PPIs were lost in the truncated transcript. In another example, the self-interacting property of the GRB2 protein was removed by the loss of domain SH2 during the exclusion of a tissue-specific exon, while its interaction to gene RAPGEF1 was retained thanks to the unscathed domain SH3. This result could be confirmed by DIGGER using the exon-view of DDI networks for GRB2 and its interacting partners. Additionally, it is possible that the rewiring events for PPIs and DDIs resulting from exon skipping will emerge from the inspection of DIGGER-generated interaction networks for different transcripts. While DIGGER currently does not offer a downstream analysis after network construction, the authors expect to expand this webservice with pathway annotation of PPIs and DDIs and a focus on investigating the biological impacts of exon skipping events.

## Detecting Protein-Protein Interaction Rewiring Events

The tools presented in the previous section enable retrieving PPIs for specific organisms, tissues, and other conditions. Yet, one is often interested in detecting changes between two conditions. For example, Basha and co-workers recently analyzed differential protein interactomes for 51 tissues from the GTEx consortium (Basha et al., 2020). In their study, they used single expression data sets and focused on establishing relationships between genes and hereditary disorders and Gene Ontology (Carbon et al., 2021) terms. The main idea behind this was to identify gene candidates to explain tissue-selectivity of these hereditary disorders.

As described in the previous sections, the aforementioned protein- and domain-level tools can be used as basis to study PPI rewiring events. However, doing so often requires custom scripts to integrate, analyze, and compare the data sets. To facilitate such differential analyses, various tools have been developed in recent years that enable scientists to detect PPI rewiring events between samples belonging to two conditions. The features of the webservices and tools presented here are summarized in Table 2.

### SPECTRA

The webtool SPECTRA (https://alpha.dmi.unict.it/spectra/) (Micale et al., 2015) provides access to tissue- and tumor-specific PPI networks and performs comparison between such networks for detection of differential interaction patterns between conditions. The tool relies on human-only protein interaction data from BioGRID (Oughtred et al., 2021), DIP (Salwinski et al., 2004), HPRD (Prasad et al., 2009), IntAct (Orchard et al., 2014), and MINT (Licata et al., 2012) to construct PPI networks. It allows users to prune the PPI network based on tissue and tumor expression profiles curated from ArrayExpress (Athar et al., 2019), the Gene Expression Omnibus (GEO) (Barrett et al., 2013), the Human Protein Atlas (Uhlén et al., 2015) and The Cancer Genome Atlas (TCGA) (Tomczak et al., 2015). Additionally, users can provide their own expression data to generate specific PPI networks for their custom defined contexts.

To identify the difference in the proteomes between multiple conditions, SPECTRA utilizes GASOLINE, a greedy and stochastic algorithm that searches for subnetworks with conserved topology between the queried PPI networks and in which the difference of expression values of aligned genes is maximized. Apart from maximizing the distance in expression levels between aligned networks, the adapted version of GASOLINE incorporates differential expression represented by log fold change into the bootstrapping phase. The differential alignments consist of sets of subnetworks sharing the same sequences and interacting patterns but differing in interaction strengths and gene expression levels. Users can either directly compare context-specific PPI networks generated by SPECTRA or upload their own sets of networks for comparison.

To illustrate how SPECTRA could assist detection and visualization of key proteins in the transition across multiple states, the authors showcase the differential alignment graph for PPI networks built using the expression data for normal, well differentiated, moderately differentiated, and poorly differentiated breast cancer tissues (Micale et al., 2015). The PPIs were taken only from BioGRID and IntAct, and each of the four PPI networks contained 7,472 nodes and 29,765 edges. The adapted GASOLINE algorithm returned 20 subnetworks, among which the largest one contained four chemokine proteins (CXCL10, CXCL9, CXCL11, CCL5), two chemokine receptors (CXCR3, CCR1) and a signal transductor DPP4. The elevated expressions of all chemokines were exhibited by increasing node sizes, which coincided with the increasing cancer grades. A similar alteration was also found in the second largest aligned hub comprised of proteins in the human leukocyte antigen (HLA) system. As the chemokine system was associated to cancer metastasis and the HLA system was reported to be responsible for immune regulation, the authors suggested that SPECTRA is a suitable tool for discovering the interactomes and differential events predisposing to tissue-specific disease.

### DifferentialNet

DifferentialNet (Basha et al., 2018) is another webservice (https://netbio.bgu.ac.il/diffnet) developed by the Yeter-Lotem lab and enables users to retrieve human tissue-specific differential protein interactions. Similar to TissueNet (Basha et al., 2017), the experimentally validated physical PPIs for DifferentialNet are taken from BioGRID (Oughtred et al., 2021), DIP (Salwinski et al., 2004), IntAct (Orchard et al., 2014), and MINT (Licata et al., 2012), while tissue expression profiles were gathered from GTEx (Lonsdale et al., 2013) and the Human Protein Atlas (Uhlén et al., 2015). To construct the differential interactome for each tissue, each protein interaction is first assigned a tissue-specific score derived from multiplying the expression levels of the two interacting proteins, thereby generating a high score for highly expressed interactions. Afterwards, the median score across tissues is computed for each interaction. The tissue-specific differential score of an interaction is then the difference between the median score and the tissue-specific score. As a result, up-regulated interactions in a given tissue receive a positive differential score, down-regulated interactions a negative differential score, and relatively unchanged interactions a differential score close to zero.

Users can select a human tissue and query DifferentialNet with up to five proteins at once. Following the concept of TissueNet, DifferentialNet then displays the tissue-specific differential interactions with the query protein(s) as an interactive graph. The interface enables users to switch between tissues or between displaying all interactions or only differential interactions, and to adjust the filtering threshold applied to the interactions on the fly. In addition, various tabs offer information about the detection method and differential scores of selected interactions or the Gene Ontology (Carbon et al., 2021) annotations and OMIM disease annotations (Amberger et al., 2019) of selected proteins. Furthermore, the complete differential interactomes can be downloaded for each tissue and have been employed for transcriptomic and network analysis studies on, for example, the association of diabetes and Alzheimer’s disease (Santiago et al., 2019), the identification of immune targets for coronary artery disease (Bon-Baret et al., 2021), and the prediction of reliable regulatory modules of colorectal cancer (Nikmanesh et al., 2020). As such, NetworkAnalyst 3.0 now includes human tissue-specific PPI data from DifferentialNet (Zhou et al., 2019).

### PPICompare

**FIGURE 3:**
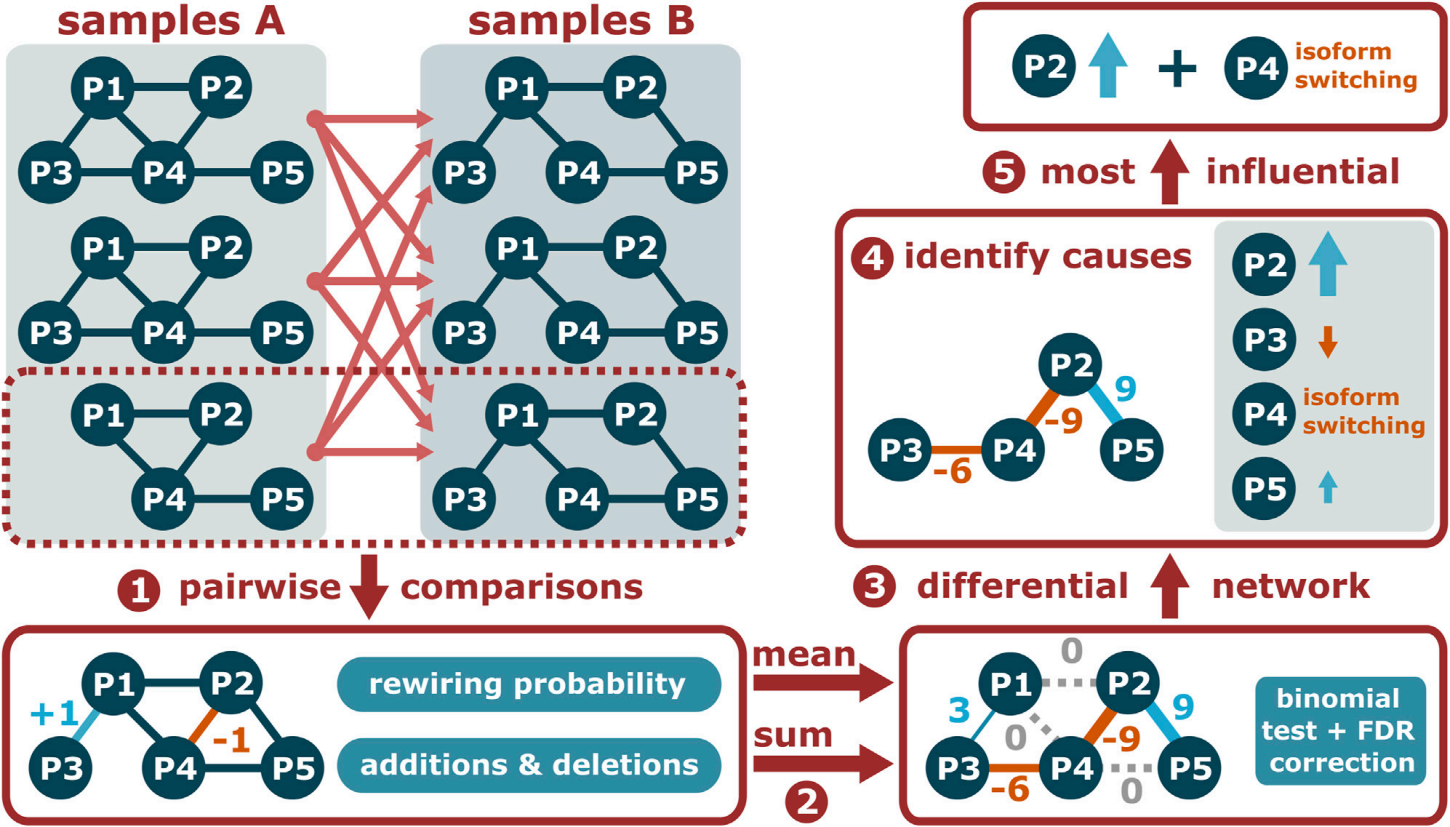
Given two sets of condition-specific PPI networks, PPICompare counts the added and deleted interactions for each pairwise comparison and the comparison-specific rewiring probability (1). Then, the overall differential PPI network is constructed by summarizing the individual comparisons (2) and identifying the statistically significant rewiring events (3). Finally, the causal reason of each rewired interaction is identified (4) and a small set of causes that can explain all of them is determined (5).

As a package to be used down-stream of PPIXpress, PPICompare (Will and Helms, 2017) is another standalone tool developed in the Helms group that identifies significantly rewired interactions between two sets of condition-specific PPI networks. Based on information contained in the input networks, it can determine the reason for each rewiring event and assembles a small set of causes that can explain all such events. Figure 3 presents an overview of the method that is further explained below.

#### Differential Protein-Protein Interaction Network Construction

First, the PPICompare tool performs independent pairwise comparisons across the two sets of condition-specific PPI networks provided as input by the user (Figure 3, step 1). In each such inter-group comparison, it is noted which interaction is added or removed in the second PPI network, and the rewiring probability is calculated as the Jaccard distance between the interaction sets of the two compared PPI networks. Subsequently, the general rewiring probability is computed as the mean of the comparison-specific rewiring probabilities, and the number of additions (positive) and removals (negative) is summed for each interaction (Figure 3, step 2). Interactions without any changes or with a null-sum, indicating a balance of additions and removals, are not included in the differential network.

Given the overall rewiring probability, the statistical significance of each potential rewiring event is determined with a one-tailed binomial test followed by false discovery rate (FDR) correction for multiple hypothesis testing with the Benjamini-Hochberg method at a user-defined threshold (Figure 3, step 3). A differential PPI network consisting of significantly rewired interactions is provided as output. If the input PPI networks contain information about the respective dominant isoform of each protein, PPICompare can additionally report for each rewiring event if it is caused by differential expression, dominant isoform switching, or possibly a combination of both (Figure 3, step 4).

On that basis, a bipartite graph of significantly rewired interactions and individual causal reasons is constructed (Figure 3, step 5). For each such reason, a score is computed from the number of significant rewiring events affected by it and the number of pairwise comparisons in which it took place. Determining a small set of causes that can explain all rewired interactions is then a weighted set-cover problem that is solved with the application of a greedy algorithm and the resulting collection of reasons is reported by the tool.

#### Application: Interactome Rewiring in Hematopoiesis

To evaluate PPICompare, a case study was performed on hematopoietic interactome rewiring (Will and Helms, 2017). First, PPIXpress was applied to 59 samples of 11 hematopoietic cell types from the BLUEPRINT epigenome project (Chen et al., 2014) to generate the cell type-specific PPI networks. The differential interactomes of adjacent cell types in the classical blood development progression model were then constructed with PPICompare and further analyzed. When comparing results on undersampled data sets, it turned out that a minimum number of three samples per group is required to yield robust results. Then, the statistical model employed by PPICompare is able extract most differentially altered interactions of possible relevance.

In most rewired interactions, differential gene expression of a single interaction partner was identified by PPICompare as the cause. A comparison with rewiring instances in which both interaction partners were deregulated showed that concurrent deregulation occurred more often in similar processes and known protein complexes, and thus possible functional modules more generally. A closer examination further indicated that different causes can be responsible for the same rewiring events. Underlining the importance of considering AS events in differential PPI analyses, alternative splicing was the identified reason for many differentially altered interactions relevant to the hematopoietic developmental transitions. Alternatively spliced proteins that were part of the set of most explanatory causes were associated with transcriptional control. Furthermore, proteins associated with hematopoiesis and targets of hematopoietic transcription factors were significantly overrepresented amongst the set of proteins participating in rewired interactions.

## Use-Case Comparison

To compare the tools from a user’s standpoint, we inspected the human tumor necrosis factor receptor protein 6 (TNR6) encoded by the FAS gene (UniProt accession: P25445) with the software tools and webservices reviewed here. Due to the large number of such tools, not all results can be discussed in detail, and we will mainly focus on the protein-level webservice HIPPIE, the domain-based webservices and tools DIIP, DIGGER and PPIXpress, and the differential network analysis tool PPICompare. An overview of all protein-level resources can be found in Table 1, while Table 2 summarizes features of the tools DIIP, DIGGER, PPIXpress, SPECTRA, DifferentialNet, and PPICompare. Figure 1 illustrates the graphical output generated by selected tools when using the human protein TNR6 as input. We compare the tools with respect to user experience and significance of results.

Differences already emerge in the input scenarios. While most protein-level resources accept query proteins, or like HIPPIE, DIIP and DIGGER additionally accept a PPI network as input, PPIXpress strictly requires a PPI network with expression data to expose condition-specific subnetworks with confidence. The integration of expression data for interaction network construction is also possible with MyProteinNet, DIGGER, and SPECTRA, although this is optional. PPICompare requires two sets of context-specific PPI and DDI networks generated by PPIXpress and is thus optimally used downstream of PPIXpress for reliable discovery of protein rewiring events caused by differential expression. For that reason, we illustrate the use of PPIXpress for the TNR6 interaction network specific for neuronal stem cell and compare the output network to a H1 stem cell-specific network using PPICompare.

The chosen tools produced results that vary in terms of information and presentation. For HIPPIE, DIGGER and PPIXpress, the visualization of network topology is available at different levels of analysis. HIPPIE, as a protein-based tool, shows a network of genes interacting with FAS where edge weights indicate interaction strength (Figure 1A). The domain-based tool DIGGER additionally shows associated domains and DDIs in the network of interacting proteins, where the edges indicate whether an interaction is missing or if residue-level evidence is available (Figure 1C). With PPIXpress, one can use Cytoscape to visualize the condition-specific weighted PPI or DDI network (Figure 1D). Results produced by PPICompare, including information for gained and lost protein interactions or the statistical significance of the rewiring event, can also be illustrated with Cytoscape (Figure 1E). As a tool that does not generate graphical visualizations for the output networks, DIIP results are summarized as look-up tables where the interactions between each isoform of the queried protein and their interacting partners are listed in long format (Figure 1B). Similarly, IID generates a table with interactions involving the query proteins and optionally a table with significantly enriched annotations, a feature that HIPPIE provides as well. While DIIP classifies an interaction only as lost or retained, all other tools provide users with a metric to assess interaction confidence (HIPPIE, SPECTRA, DifferentialNet, and PPIXpress) or domain-based reliability of the found PPIs (DIGGER and PPICompare).

As a consequence of differences in the underlying data sources, methodologies and focuses, the results for TNR6 differ in terms of the number of found interactions and the type of information. For example, HIPPIE found 118 PPIs involving TNR6, while IID found 367 including predicted interactions and 122 when only considering experimentally validated ones. Both computed significant enrichment of proteins associated with cancer among the interaction partners. In contrast, DIIP found two experimentally observed interactions with underlying DDIs for TNR6, namely with the tumor necrosis factor ligand 6 (TNFL6, UniProt accession: P48023) and the FAS-associated death domain protein (FADD, UniProt accession: Q13158). It predicted that for all but one TNR6 isoform the interaction with FADD would be lost, whereas most isoforms can enter domain-based interactions with TNFL6. DIGGER additionally offers isoform- and exon-level visualization of the interacting domains. It is thus possible to visually inspect how the domain presence leads to the loss or retention of the protein interaction in TNR6 isoforms predicted by DIIP. A similar analysis can be performed based on the results produced by PPIXpress but requires users to manually visualize and overlay the generated PPI and DDI networks. While comparing sample-specific PPI and DDI networks generated by PPIXpress from H1 stem cells to those from neuronal stem cells, PPICompare detected two statistically significant rewiring events (*p* < 0.01) involving TNR6. In neuronal stem cells, TNR6 gains interactions with FAIM2 (UniProt accession: Q9BWQ8) and TCAP (UniProt accession: O15273). FAIM2 is an important regulatory molecule for apoptotic control during neurological development and pathogenesis (Reich et al., 2011), while TCAP was associated to dendrite and axon formation during neurogenesis (Woelfle et al., 2016).

In summary, the results of the reviewed tools are diverse and suggest that users should weigh the advantages and disadvantages of each method carefully for the purpose of their specific needs. Depending on the use-case, it may be beneficial to cross-check and compare the results from multiple tools to obtain a richer picture.

## Outlook

In this final section, we discuss some technical points of the presented methods and where we see a potential for future improvements. In addition, we point out future directions and areas of applications of context-specific PPI networks.

### Use of Proteomic Data Instead of Transcriptomic Data

First, one may wonder why context-specific PPI networks are typically constructed based on transcriptomic data rather than on proteomic data from mass spectrometry. Experimentally measured mRNA and protein concentrations typically show an average Pearson correlation of about 0.6 in mammalian tissues (Buccitelli and Selbach, 2020) and the true correlation is expected to be higher than this. However, there are of course clear outliers that may occur if mRNA and protein half-lives differ substantially. The reason for working with transcriptomic data is simply the scarcity of available proteomic data, as publications often only report at most a few mass spectrometry datasets.

The overall scarcity of proteomic data and frequent lack of replicate data sets is particularly problematic for approaches that aim to determine PPI rewiring events in a statistically significant manner (Will and Helms, 2017). A first step in this direction was made by Liu et al. who used proteome data from mass spectrometry for 30 tissues to generate tissue-specific PPI networks based on experimental data for protein concentrations (Liu et al., 2014). The authors realized the problematic nature of single data sets and tried to reduce this effect by only considering the overlap of two experimental studies. This gave 627 tissue specific proteins and 1,093 housekeeping proteins, which is likely a low estimate of the actual numbers. A remarkable step forward was made recently in a proteomic atlas of human skin that reported 3–5 mass spectrometry data sets each for CD1A+dendritic cells, CD14^+^ dendritic cells, macrophages, mast cells, fibroblast, keratinocytes, endothelial cells, melanocytes, and skin layers (Dyring-Andersen et al., 2020). An increased prevalence of similar datasets would likely lead to an increased usage of proteomic data in PPI rewiring studies and stimulate the adaptation and development of existing and new PPI network software tools and webservices.

While transcriptomic data is much more abundantly available than proteomic data, there are nevertheless research areas where typically only a few replicates are measured. Pooling transcriptomic data from different studies could enable the study of PPI network rewiring in these cases. However, it remains to be carefully examined how robust PPI networks are with respect to the technical variability introduced by combining multiple sources.

### Technical Aspects of Working With Transcriptomic Data

Context-specific PPI network approaches use gene expression levels to approximate which PPIs are present in the given condition. A technical question is then what concentration level should be used to consider a gene as “sufficiently expressed” so that the respective protein is available to form interactions. The most straightforward approach is to simply take all genes covered by at least a single read. Another option is to select and justify applying a minimum threshold. A suitable threshold could, for example, be derived from independent proteome abundance data (Will and Helms, 2017). However, the underlying proteomics experiments use thresholds as well, and it is not clear if relying on those leads to an improvement.

### Single Cell Transcriptomic Datasets

Recently, there has been a considerable shift of attention in the transcriptomic field toward single-cell sequencing. So far, we detected only a single study where such data was used to infer cell-type specific protein interactomes. Namely, Kellis and co-workers recently extended the expression-level filtering concept to single cell RNAseq data and attempted to construct PPI networks for single cells (Mohammadi et al., 2019). Due to the noisy nature of scRNAseq data, they applied regression-based imputation to infer missing values and to balance gene expression levels. Given the increased availability of such data sets, there is certainly a considerable potential for such applications in future.

### Extending Domain-Level Approaches

Domain- and isoform-level approaches such as DomainGraph, DIIP, DIGGER, PPIXpress, and PPICompare use Pfam domains as smallest units for resolving alternative splicing effects. Under this paradigm, about half of all human proteins can be assigned to one or more Pfam domains. However, this approach does not allow resolving AS events that affect smaller splice insertions or deletions. Hence, it appears worthwhile to extend the description of domains by also including shorter amino acid sequences such as, for example, short linear motifs. While these motifs often occur in structurally disordered regions, they are contiguous amino acid modules that nevertheless show evolutionary conservation (Kumar et al., 2020) and can be specified and identified based on regular expressions (Davey et al., 2007; Diella et al., 2008; Van Roey et al., 2014). They have been shown to be relevant for interaction rewiring by splicing (Yang et al., 2016) and may thus extend the applicability of domain-level approaches to model interactions mediated by such regions that often play crucial roles in cellular control mechanisms (Buljan et al., 2012; Uversky, 2018). There already exist established resources on short motifs such as the ELM (eukaryotic linear motif) database (Kumar et al., 2020) as well as more general approaches working on short peptides (Chen et al., 2015) that could prove valuable for future inclusion.

### Data Integration

Apart from modelling the rewiring of interactions due to changes in the transcriptome, it is also of considerable interest to study how protein mutations affect the edge-specific adaption of the interactome (Zhong et al., 2009; Sahni et al., 2013; Sahni et al., 2015). Whereas established tools such as SIFT (Kumar et al., 2009) and PolyPhen2 (Adzhubei et al., 2010) estimate whether a mutation affects the general function of a protein, recent tools that rely on structural data such as dSysMap (Mosca et al., 2015) and StructMAn (Gress et al., 2016) attempt to infer the effect of mutations on specific protein interactions. Furthermore, the IMEx Consortium recently released a data set of roughly 28,000 mutations and their effects on physical protein interactions that was curated from experimental data (Del-Toro et al., 2019).

It further appears worthwhile to connect the rewiring analysis of PPI networks to alterations in other data types. For example, one could inspect formation of protein complexes involving chromatin reader or modifier proteins and relate this to respective epigenetic data on histone marks. Additionally, one could correlate the presence or absence of specific protein isoforms to the activity of individual splicing factors that are predicted to bind at the respective exon-intron boundaries. Splicing factor activity can either by modified by deregulated expression or by the presence or absence of epigenetic marks at exon/intron boundaries.

Finally, one may also connect PPI networks to gene-regulatory networks and connect, for example, the combinatorial complexity of transcription factor complexes to the presence or absence of their transcription factor binding motifs in the promoters of their target genes. This was done for transcription factor-containing protein complexes of yeast that were then related to their activity in regulating target genes that periodically cycle during the cell-cycle of yeast (Will and Helms, 2014).

## Conclusion

In summary, there are now several sophisticated tools available to construct context-specific protein-protein interaction networks. These include both webservices as well as stand-alone software packages, some of which support domain-level and isoform-level analyses. Differential analysis of context-specific PPI networks is currently only possible with the tools SPECTRA, DifferentialNet and PPICompare. SPECTRA and DifferentialNet are available as easily accessible web-based resources for human PPIs. On the other hand, the stand-alone tool PPICompare more generally enables identifying statistically significant rewiring events between two groups of samples. Lastly, integration with other data types appears worthwhile.
